# Long-term effect of feeding snacks at age 6 years on body mass index at ages 12 and 22 years

**DOI:** 10.1038/s41598-019-40730-3

**Published:** 2019-06-13

**Authors:** Mizuki Sata, Kazumasa Yamagishi, Toshimi Sairenchi, Ai Ikeda, Fujiko Irie, Hiroshi Watanabe, Hiroyasu Iso, Hitoshi Ota

**Affiliations:** 1 Public Health, Department of Social Medicine, Osaka University Graduate School of Medicine, Osaka, Japan; 2 Ibaraki Health Plaza, Ibaraki, Japan; 3 Department of Preventive Medicine and Public Health, Keio University School of Medicine, Tokyo, Japan; 4 Department of Public Health Medicine, Faculty of Medicine, University of Tsukuba, Ibaraki, Japan; 5 Ibaraki Health Service Association, Ibaraki, Japan; 6 Department of Public Health, Dokkyo Medical University School of Medicine, Tochigi, Japan; 7 Department of Public Health, Juntendo University School of Medicine, Tokyo, Japan; 8 Department of Health and Welfare, Ibaraki Prefectural Office, Ibaraki, Japan

**Keywords:** Lifestyle modification, Epidemiology, Risk factors

## Abstract

We investigated the effect of snacking habits in childhood on changes in body mass index (BMI) and high BMI in adolescence and adulthood. In total, 2141 Japanese children from the Ibaraki Children’s Cohort Study were evaluated at age 6 years (baseline), then at ages 12 and 22 years. We examined associations between snacking (scheduled times, when children wanted, and freely) at age 6 years and changes in BMI over time and the proportion of high BMI at ages 12 and 22 years, using time-dependent mixed-effects and logistic regression models. Compared with children who snacked at scheduled times, those provided snacks when they wanted experienced larger increases in BMI over time between ages 6 and 22 years (multivariable time-dependent effect: 0.03 kg/m^2^ for boys, *p* = 0.047; 0.04 kg/m^2^ for girls, *p* = 0.019). No differences were observed in children who snacked freely. A higher proportion of high BMI was found in boys who were provided snacks when they wanted compared with those who snacked at scheduled times. The multivariable odds ratio (95% confidence interval) was 1.52 (1.04–2.23) at age 12 years and 2.23 (1.12–4.45) at age 22 years. No differences were observed for girls at either age. Children who were provided snacks when they wanted showed larger increases in BMI over time compared with those who snacked at scheduled times. Boys who were provided snacks when they wanted showed the higher proportion of high BMI at follow-up.

## Introduction

Obesity among children and young adults is an emerging health problem worldwide^1^. In Japan, the proportions of obesity (obesity index ≥ 20%) among boys and girls aged 11 years were 9.7% and 8.7%, respectively^2^. In 2017, the proportions of men and women aged 20–29 years with obesity (defined as a body mass index [BMI] ≥ 25.0 kg/m^2^) were 26.8% and 5.7%, respectively^3^. Although these proportions were not as high in Eastern countries as in Western countries such as the US, rapid Westernization may result in increased proportions of obesity. In turn, this may increase the burden of diabetes, hypertension, and atherosclerotic diseases^4^. In addition to physical inactivity, eating behaviors may play an important role in obesity^5^.

Although snacking, which is characterized by eating smaller amounts of foods other than three regular meals a day, contributes to the intake of important micronutrients in children^6^, previous case-control^7^ and cross-sectional^8^ studies showed that the frequency of snacking was associated with consumption of energy-dense foods and an increased proportion of obesity in children and adolescents. To date, one cross-sectional study^9^ and two Japanese cohort studies^10,11^ have shown that consuming snacks without time constraints was associated with risk for developing obesity in childhood and adolescence. Parenting affects children’s behavior leading to obesity because caregivers’ behaviors (e.g., feeding snacks) are determined by their recognition of their children’s attitudes toward hunger and satiety^12^. However, several cross-sectional studies showed that the frequency of snacking was weakly and inversely associated with the proportion of obesity for children aged 3–19 years^13,14^. The effect of consuming snacks in childhood on subsequent obesity in young adulthood and adolescence is unclear because no previous cohort studies have investigated the long-term effect.

This cohort study aimed to examine the associations between snacking habits at age 6 years and changes in BMI and the proportion of high BMI at ages 12 and 22 years. Our *a priori* hypothesis was that children who are provided snacks when they want or those who snack freely at age 6 years experience a larger increase in BMI and are more likely to have high BMI later in life compared with those who are provided snacks at scheduled times.

## Methods

### Participants

The Ibaraki Children’s Cohort (IBACHIL) Study is a long-term prospective cohort study involving children born in 1989 in 87 communities in Ibaraki Prefecture, Japan. In 1992, we distributed a health questionnaire to parents at the site of a community-based health checkup for 3-year-old children (n = 4592). Follow-up surveys were conducted when the children were aged 6 (n = 2141; follow-up rate, 47%), 12 (n = 2375; follow-up rate, 52%), and 22 (n = 1559; follow-up rate, 34%) years based on mail-in surveys. At ages 3, 6, and 12 years, respondents were the children’s parents. At age 22 years, the respondents were the study subjects themselves. We excluded 125 children from the sample because: (1) they were unaccustomed to eating snacks when the questionnaire was provided at age 6 years (n = 43), (2) information was incomplete about frequency of snaking/snack feeding for children when the questionnaire was provided at age 6 years (n = 106), and (3) data was missing regarding sex (n = 1). Finally, 2016 children (1072 boys and 944 girls) were included in this analysis (94.2%).

Return of a completed questionnaire for children at ages 3, 6, and 12 years with signatures of children by parents (per procurationem) was regarded as provision of informed consent. At age 22 years, we obtained informed consent from the participants. All procedures were performed in accordance with the “Ethical Guidelines for Epidemiological Research” and “Ethical Guidelines for Medical and Health Research Involving Human Subjects.” The study protocol was approved by the Epidemiology Combination Ethics Review Committee of Ibaraki Prefecture.

### Baseline measurements

The questionnaire administered to parents regarding their 6-year-old children covered several physical and lifestyle factors. Snacking behavior for children was discerned from the original (baseline) questionnaire at age 6 years, which classified children as: (1) being provided snacks at scheduled times, (2) being provided snacks under parents’ control when children wanted, and (3) snacking freely (not under parents’ control). Other items included: child’s height and weight; time for play outside on week days (less than 1 hour, approximately 1 hour, approximately 2 hours, approximately 3 hours, or ≥4 hours); habit of speed-eating (yes, somewhat, rarely, or never); frequency of breakfast (every day, five to six times a week, three to four times a week, one to two times a week, or never); between-meal eating behavior 30 minutes before dinner (feeding light foods or keeping the child waiting); frequency of between-meal eating before bedtime (every day, three to five times a week, one to two times a week, two to three times a month, once a month, or never); paternal and maternal height and weight; paternal job (full-time job, part-time job, independent business, agriculture, forestry and fisheries, unemployed, night shift,  or others); and maternal job (full-time job, part-time job, independent business, agriculture, forestry and fisheries, full-time housewife, unemployed, or others).

We defined “less than 2 hours of playing outside” as physical inactivity. Speed-eating was defined by responses of “yes” or “somewhat,” and skipping breakfast by responses of “five to six times a week,” “three to four times a week,” “one to two times a week,” “or never” as skipping breakfast. “Feeding light foods” was considered between-meal eating before dinner, and “everyday” or “three to five times a week” was considered between-meal eating before bedtime. Both paternal and maternal employment were defined by “full-time job.” The child’s BMI was calculated as their weight (kg) divided by the square of their height in meters (m^2^) based on questionnaire data. High BMI was defined as a BMI ≥16.9 kg/m^2^ for boys and ≥17.1 kg/m^2^ for girls at age 6 years (≥1 standard deviation of the World Health Organization sex- and age-specific BMI distributions for children)^15^.

### Follow-up measurements

Similar questionnaires were distributed when children were aged 12 and 22 years. Data for height and weight were missing for 636 children at age 12 years (29.6% of the 6-year-old sample) and 1090 (50.9%) at age 22 years. At age 12 years, high BMI was defined as a (questionnaire-based) BMI of ≥20.4 kg/m^2^ for boys and ≥21.3 kg/m^2^ for girls^15^. At age 22 years, high BMI was ≥25.0 kg/m^2^ for boys and girls (corresponding to the adult criteria).

### Statistical analyses

Differences in mean values or frequencies of baseline characteristics according to snacking habits for children (provided snacks when they wanted or snacking freely vs. provided snacks at scheduled times) were tested by analysis of variance. Chi-square tests were used to evaluate differences in proportions between responders and non-responders at ages 12 and 22 years.

An analysis of repeated measures for BMI was performed using time-dependent mixed-effects models to examine the impact of snacking behaviors on BMI over time. This accounted for the correlation between repeated measurements taken from the same individual^16^. In the present study, we used the following model.

$$\begin{array}{rcl}yij & = & ({\rm{\beta }}0+{\rm{b}}0i)+{\rm{\beta }}p\cdot Snacking2i+{\rm{\beta }}p\cdot Snacking3i+{\rm{\beta }}T\cdot timeij\\  &  & +{\rm{\beta }}PT\cdot (Snacking2\times time)ij+{\rm{\beta }}PT\cdot (Snacking3\times time)ij+\varepsilon ij\end{array}$$

where *yij* represents the individual’s BMI *i* taken at time *j*; β0 represents the intercept, and β*T* the slope of the linear relationship between the BMI and time; β*P* represents the effect of snacking behavior on BMI (considered as constant across time); snacking2 represents the snacking behavior of being provided snacks when children wanted; snacking3 represents the snacking behavior of snacking freely; and β*PT* represents the effect of snacking behavior on the slope, describing the linear relationship between BMI and time. Coefficients for this model were estimated by the maximization of likelihood using the SAS procedure MIXED, and specifying a compound symmetry structure for the covariance matrix. To examine whether snacking behavior and associations with BMI were affected by potential confounders that were related to snacking habits and each outcome and parental characteristics, we adjusted for physical inactivity (yes or no), speed-eating (yes or no), skipping breakfast (yes or no), between-meal eating before dinner (yes or no), between-meal eating before bedtime (yes or no), paternal employment (yes or no), maternal employment (yes or no), paternal BMI (<18.5 kg/m^2^, 18.5–24.9 kg/m^2^, or ≥25 kg/m^2^), and maternal BMI (<18.5 kg/m^2^, 18.5–24.9 kg/m^2^, or ≥25 kg/m^2^).

We used logistic regression models to calculate sex-specific odds ratios (ORs) and 95% confidence intervals (CIs) for high BMI at ages 12 and 22 years according to children’s snacking habits. These models were adjusted for children’s baseline BMI category (quintiles) for ages 12 and 22 years, and BMI difference categories (quintiles) between ages 6 and 12 years for age 22 years. The other potential confounding factors were also included in multivariate logistic regression models.

All statistical analyses were performed with SAS version 9.4 (SAS Institute, Inc., Cary, NC, USA). All probability values for statistical tests were two-tailed, and *p-*values < 0.05 were regarded as statistically significant.

## Results

At age 6 years, 51.1% (51.1% of boys, 51.1% of girls) of children were provided snacks at scheduled times, 41.6% (43.1% of boys, 39.8% of girls) were provided snacks when they wanted, and 7.3% (5.8% of boys, 9.1% of girls) snacked freely. The mean baseline BMI and proportions of high BMI (boys only), inactivity (boys only), skipping breakfast, between-meal eating before dinner, and between-meal eating before bedtime (boys only) were higher in children who were provided snacks when they wanted compared with those who were provided snacks at scheduled times (Table 1). The proportions of speed-eating (boys only), skipping breakfast, between-meal eating before dinner, and between-meal eating before bedtime were higher in children who snacked freely compared with those who were provided snacks at scheduled times. Paternal employment was significantly lower and maternal employment was higher among children who snacked freely compared with those who snacked at scheduled times. The mean paternal BMI value was significantly higher among girls who were provided snacks when they wanted and snacked freely compared with those who were provided snacks at scheduled times. However, maternal BMI did not differ by snacking habits.

**Table 1:** Sex-specific means ± standard deviations and proportions for baseline characteristics at age 6 years among 1072 boys and 944 girls (IBACHIL, 1995).

Snacking behavior for 6-year-old children	Boys			Girls		
	Being provided at schedule times	Being provided when children wanted	Eating freely	Being provided at schedule times	Being provided when children wanted	Eating freely
	(n = 548)	(n = 462)	(n = 62)	(n = 482)	(n = 376)	(n = 86)
BMI, kg/m^2^	15.9 ± 1.6	16.2 ± 2.0*	16.3 ± 1.5	15.7 ± 1.7	16.1 ± 1.7*	15.8 ± 1.8
High BMI, %	17.0	25.8**	28.3	15.0	18.2	12.1
Inactivity (playing outside < 2 hours on weekdays), %	25.9	32.4*	26.2	28.5	34.6	36.1
Speed-eating, %	45.6	50.3	65.6**	28.1	34.0	36.9
Skipping breakfast (eating breakfast ≤6 times per week), %	6.0	13.2***	21.0***	6.7	12.3*	16.7**
Between meal eating before dinner, %	33.5	63.9***	72.6***	32.9	57.3***	69.0***
Between meal eating before bedtime ≥3 times per week, %	7.5	12.9*	19.7**	7.3	11.8	17.4**
Paternal employment, %	86.1	81.2	72.1*	83.5	81.3	72.3*
Paternal BMI, kg/m^2^	23.1 ± 2.9	23.2 ± 2.7	23.5 ± 2.8	23.0 ± 2.8	23.6 ± 2.8*	24.0 ± 2.6*
Maternal employment, %	14.9	16.4	24.2	12.5	17.4	26.7**
Maternal BMI, kg/m^2^	21.1 ± 2.5	21.4 ± 2.6	21.6 ± 2.4	20.9 ± 2.4	21.2 ± 2.5	21.2 ± 2.5

BMI, body mass index.

*p < 0.05, **p < 0.01, ***p < 0.001, compared with children who were provided snacks at scheduled times at age 6 years.

There were no sex-specific differences in mean BMI values and proportions of high BMI at age 6 years between the responders and non-responders at ages 12 and 22 years, except for a difference in the proportion of high BMI among boys between the responders and non-responders at age 22 years (responders 18.4% vs. non-responders 24.3%, *p* = 0.044) (Supplementary Table).

Table 2 shows sex-specific changes in BMI over time according to snacking behavior at age 6 years. Children who were provided snacks when they wanted experienced a larger increase in BMI over time between ages 6 and 22 years compared with children who snacked at scheduled times. The multivariable time-dependent effect was 0.03 kg/m^2^ (*p* = 0.047) for boys and 0.04 kg/m^2^ (*p* = 0.019) for girls. However, there was no significant difference in BMI over time between children who snacked freely and those who were provided snacks at scheduled times. The multivariable time-dependent effect was 0.02 kg/m^2^ (*p* = 0.480) for boys and 0.04 kg/m^2^ (*p* = 0.180) for girls.

**Table 2:** Sex-specific and multivariable adjusted changes in BMI over time according to children’s snacking behavior at age 6 years.

	Boys							Girls						
	Snacking behavior for 6-year-old children							Snacking behavior for 6-year-old children						
	Being provided at schedule times	Being provided when children wanted			Eating freely			Being provided at schedule times	Being provided when children wanted			Eating freely		
	*β*	*β*	95% CI	*p* value	*β*	95% CI	*p* value	*β*	*β*	95% CI	*p* value	*β*	95% CI	*p* value
Crude model														
Baseline effect	0	0.41	0.06–0.77	0.021	0.37	−0.36–1.11	0.321	0	0.44	0.10–0.79	0.012	0.07	−0.54–0.67	0.827
Time-dependent effect	0	0.03	−0.003–0.06	0.073	0.02	−0.04–0.09	0.511	0	0.04	0.002–0.07	0.036	0.04	−0.02–0.09	0.208
Multivariable model^a^														
Effect at baseline	0	0.39	0.03–0.75	0.035	0.20	−0.54–0.95	0.594	0	0.36	0.005–0.71	0.047	−0.10	−0.72–0.53	0.759
Time-dependent effect	0	0.03	−0.0004–0.06	0.053	0.02	−0.04–0.08	0.524	0	0.04	0.003–0.07	0.031	0.04	−0.02–0.10	0.178
Multivariable model^b^														
Effect at baseline	0	0.35	−0.010–0.70	0.057	−0.02	−0.75–0.72	0.961	0	0.27	−0.07–0.62	0.121	−0.17	−0.78–0.45	0.593
Time-dependent effect	0	0.03	0.0005–0.06	0.047	0.02	−0.04–0.09	0.480	0	0.04	0.007–0.08	0.019	0.04	−0.02–0.10	0.180

BMI, body mass index; CI, confidence interval.

^a^Multivariable models adjusted for playing outside, speed-eating, skipping breakfast, between-meal eating before dinner, and between-meal eating before bedtime at age 6 years.

^b^Further adjusted for paternal employment, maternal employment, paternal BMI, and maternal BMI at age 6 years.

The multivariable ORs (95% CI) for high BMI at age 12 years among children who were provided snacks when they wanted compared with those who were provided snacks at scheduled times were 1.52 (1.04–2.23) for boys and 1.15 (0.69–1.92) for girls (Table 3). The corresponding multivariable ORs (95% CI) for high BMI at age 12 years for children who snacked freely were 0.81 (0.36–1.82) and 1.01 (0.41–2.51), respectively. The multivariable ORs (95% CI) for high BMI at age 22 years among children who were provided snacks when they wanted compared with those who were provided snacks at scheduled times were 2.23 (1.12–4.45) for boys and 0.97 (0.38–2.51) for girls. The corresponding multivariable ORs (95% CI) for high BMI at age 22 years for children who snacked freely were 3.29 (1.09–9.92) and 1.74 (0.37–8.27), respectively.

**Table 3:** Sex-specific and multivariable adjusted odds ratios for high BMI at ages 12 and 22 years according to children’s snacking behavior at age 6 years.

Snacking behavior for 6-year-old children	Number of subjects	Number of cases	Baseline BMI- adjusted ORs^a^	Multivariable- adjusted ORs^b^	Multivariable- adjusted ORs^c^
**High BMI at age 12 years**					
Boys					
Being provided at schedule times	368	75	1.0	1.0	1.0
Being provided when children wanted	347	104	1.62 (1.14–2.29)	1.53 (1.05–2.22)	1.52 (1.04–2.23)
Eating freely	47	10	1.04 (0.49–2.22)	0.85 (0.38–1.88)	0.81 (0.36–1.82)
Girls					
Being provided at schedule times	317	48	1.0	1.0	1.0
Being provided when children wanted	248	43	1.18 (0.75–1.88)	1.14 (0.70–1.86)	1.15 (0.69–1.92)
Eating freely	53	8	1.04 (0.45–2.38)	1.05 (0.44–2.50)	1.01 (0.41–2.51)
**High BMI at age 22 years**					
Boys					
Being provided at schedule times	241	18	1.0	1.0	1.0
Being provided when children wanted	222	31	1.88 (1.01–3.50)	2.11 (1.09–4.07)	2.23 (1.12–4.45)
Eating freely	30	8	3.71 (1.41–9.71)	3.93 (1.37–11.2)	3.29 (1.09–9.92)
Girls					
Being provided at schedule times	232	14	1.0	1.0	1.0
Being provided when children wanted	162	12	1.10 (0.49–2.51)	1.13 (0.46–2.78)	0.97 (0.38–2.51)
Eating freely	39	3	1.44 (0.38–5.54)	1.49 (0.32–6.90)	1.74 (0.37–8.27)

BMI, body mass index; CI, confidence interval; OR, odds ratio.

^a^Adjusted for baseline children’s BMI category for ages 12 and 22 years; and the category of BMI difference between ages 6 and 12 years for age 22 years.

^b^Multivariable models adjusted for baseline BMI, playing outside, speed-eating, skipping breakfast, between-meal eating before dinner, and between-meal eating before bedtime at age 6 years.

^c^Further adjusted for paternal employment, maternal employment, paternal BMI, and maternal BMI at age 6 years.

## Discussion

In this prospective study, boys and girls who were provided snacks when they wanted in early childhood experienced a slightly larger increase in BMI over time compared with those who were provided snacks at scheduled times. Further, we found that boys who were provided snacks when they wanted in early childhood later showed a higher proportion of high BMI in adolescence and adulthood. Boys who snacked freely in early childhood later only showed a higher proportion of high BMI in adulthood.

In a case-control study involving 100 Brazilian children aged 6–8 years, the frequent intake of snacks (high-energy foods such as fried salty foods and soft drinks) was associated with a higher proportion of obesity (OR = 10.4; 95% CI: 1.3–83.9)^7^. In a cross-sectional study of 10,684 US children and adolescents aged 6–19 years, snack intake based on percentage contribution to total energy intake was also associated with a higher proportion of obesity (OR = 1.19; 95% CI: 1.06–1.34 in children; OR = 1.16; 95% CI: 1.04–1.29 in adolescents)^8^. Furthermore, two Japanese cohort studies showed associations between snacking habits in childhood and obesity in adolescence. In a study of 737 children, the multivariable OR (95% CI) for obesity (Kaup index ≥18) in children aged 9–13 years who consumed snacks without time constraints at age 3 years was 2.12 (1.25–3.61) compared with those who were provided snacks at scheduled times^10^. In another study involving 6762 children, the multivariable OR (95% CI) for obesity at age 9–10 years for those who ate snacks irregularly at age 3 years was 1.75 (1.29–2.37) compared with those who ate snacks regularly^11^. These results were consistent with our findings in adolescents.

The reason for a lack of association between snacking freely at age 6 years and high BMI at age 12 years is uncertain. A habit of eating freely at ages 6 and 12 years was reported by parents, while that at age 22 years was reported by the child. Parental recognition of a habit of snacking freely may not reflect the actual snacking situation, as parents may not know if a child has snacked or not. Young children generally do not have much access to eat snacks freely outside the home.

Changes in BMI over multiple measurements across time and the proportion of high BMI in adolescence/adulthood were larger among boys who were provided snacks when they wanted than among those were provided snacks at scheduled times. For girls, we found a similar result for changes in BMI, but not for high BMI. In part, this may be explained by the smaller number of girls with high BMI.

A strength of this study was that the long-term prospective design enabled observation of prolonged lifestyle habits from ages 6 to 22 years. To our knowledge, this is the first longitudinal study to examine the associations between parents’ snack feeding style in childhood with changes in BMI over time and high BMI in adolescence and adulthood.

The present study had several limitations. First, information such as children’s height, weight, and snacking behavior were derived from self-administered questionnaires completed by their parents. In a previous study of adolescents aged 8–12 years, parent-reported weight was understated by 0.671 kg for boys and 1.274 kg for girls, and parent-reported height was understated by 0.504 cm for boys and 1.262 cm for girls; BMI based on parent-reported data was therefore understated by 0.200 kg/m^2^ for boys and 0.251 kg/m^2^ for girls^17^. In another study involving adults, self-reported weight was understated by 1.6% for men and 3.1% for women, whereas height was overstated by 1.3% for men and 0.6% for women^18^. Therefore, misclassification of self-reported BMI data in our study may be small. Second, the follow-up rate from the baseline survey (at age 6 years) was not high (68.5% at 12 years and 45.9% at 22 years). This may be attributable to participants moving to other prefectures to seek higher education or an occupation. However, mean BMI and the proportion of high BMI at baseline did not differ substantially between the responders and non-responders at ages 12 and 22 years, except for the proportion of high BMI for boys between the responders and non-responders at age 22 years. Because of the potential underrepresentation of high BMI status, the real association between feeding snacks and the proportion of high BMI for boys at age 22 years may be stronger. Third, we did not have detailed dietary information (e.g., energy from snacks), which might have confounded the associations we observed. Finally, we did not collect detailed information on socioeconomic status. However, we had information on parental occupation, and the associations between being provided snacks when children wanted with increasing BMI and the proportion of high BMI remained significant after adjustment for parental employment.

In conclusion, both boys and girls who were provided snacks when they wanted showed a slight but larger increase in BMI over time compared with those who were provided snacks at scheduled times. Further, boys who snacked in such manner in early childhood later showed a higher proportion of high BMI in adulthood.

## Supplementary information

Supplementary Table
