# Supplementary material for: Long-term effect of feeding snacks at age 6 years on body mass index at ages 12 and 22 years
Source: Sci Rep. 2019 Jun 13;9:8627. doi: 10.1038/s41598-019-40730-3 (PMC6565708; doi:10.1038/s41598-019-40730-3)
Supplement: Supplementary file 1 — Supplementary Table [file 41598_2019_40730_MOESM1_ESM.pdf]

Supplementary Information

Long-term effect of feeding snacks at age 6 years on body mass index at ages 12 and 22 years

Mizuki Sata[1,2,3,4], Kazumasa Yamagishi[2,4,5], Toshimi Sairenchi[2,5,6], Ai Ikeda[7], Fujiko Irie[8], Hiroshi Watanabe[5], Hiroyasu Iso[1,4] & Hitoshi Ota[2,5]

1. Public Health, Department of Social Medicine, Osaka University Graduate School of Medicine, Osaka, Japan.
2. Ibaraki Health Plaza, Ibaraki, Japan.
3. Department of Preventive Medicine and Public Health, Keio University School of Medicine, Tokyo, Japan.
4. Department of Public Health Medicine, Faculty of Medicine, University of Tsukuba, Ibaraki, Japan.
5. Ibaraki Health Service Association, Ibaraki, Japan.
6. Department of Public Health, Dokkyo Medical University School of Medicine, Tochigi, Japan.
7. Department of Public Health, Juntendo University School of Medicine, Tokyo, Japan.
8. Department of Health and Welfare, Ibaraki Prefectural Office, Ibaraki, Japan.

Supplementary Table. Sex-specific means ±standard deviations of BMI and proportions of high BMI at age 6 years between the responders and non-responders.

	Boys			Girls		
	Responders	Non-responders	*p* for difference	Responders	Non-responders	*p* for difference
At age 12 years						
Mean ± standard deviations of BMI at ages 6 years, kg/m^2	16.0 ±1.7	16.0 ±1.9	0.908	15.9 ±1.7	15.8 ±1.6	0.373
High BMI at age 6 years, %	22.6	17.8	0.161	17.1	13.6	0.250
At age 22 years						
Mean ± standard deviations of BMI at ages 6 years, kg/m^2	15.9 ±1.6	16.1 ±1.9	0.148	15.8 ±1.8	15.9 ±1.6	0.833
High BMI at age 6 years, %	18.4	24.3*	0.044	16.7	15.5	0.679

BMI, body mass index.

*$p < 0.05$, compared with responders at ages 12 or 22 years.
